# Machine learning in dental, oral and craniofacial imaging: a review of recent progress

**DOI:** 10.7717/peerj.11451

**Published:** 2021-05-17

**Authors:** Ruiyang Ren, Haozhe Luo, Chongying Su, Yang Yao, Wen Liao

**Affiliations:** 1 State Key Laboratory of Oral Diseases & National Clinical Research Center for Oral Diseases, West China School of Stomatology, Sichuan University, Chengdu, Sichuan, China; 2 School of Computer Science, Sichuan University, Chengdu, Sichuan, China; 3 State Key Laboratory of Oral Diseases & National Clinical Research Center for Oral Diseases, Department of Implantology, West China Hospital of Stomatology, Sichuan University, Chengdu, Sichuan, China; 4 State Key Laboratory of Oral Diseases & National Clinical Research Center for Oral Diseases, Department of Orthodontics, West China Hospital of Stomatology, Sichuan University, Chengdu, Sichuan, China; 5 Department of Orthodontics, Osaka Dental University, Hirakata, Osaka, Japan

**Keywords:** Orthodontics, Oral cancer, Machine learning, Dental, oral and craniofacial imaging

## Abstract

Artificial intelligence has been emerging as an increasingly important aspect of our daily lives and is widely applied in medical science. One major application of artificial intelligence in medical science is medical imaging. As a major component of artificial intelligence, many machine learning models are applied in medical diagnosis and treatment with the advancement of technology and medical imaging facilities. The popularity of convolutional neural network in dental, oral and craniofacial imaging is heightening, as it has been continually applied to a broader spectrum of scientific studies. Our manuscript reviews the fundamental principles and rationales behind machine learning, and summarizes its research progress and its recent applications specifically in dental, oral and craniofacial imaging. It also reviews the problems that remain to be resolved and evaluates the prospect of the future development of this field of scientific study.

## Introduction

Artificial Intelligence (AI) has been one of the most popular realms of scientific research in the past few decades and plays a role in the daily lives of many people (Abhimanyu et al., 2020; Dolci, 2017; Wang et al., 2020; Yanhua, 2020). AI is the ability of computers to learn from the input of data. It aims to find an optimal and adaptive approach to solve problems flexibly without the help of human beings (Legg & Hutter, 2007; Visvikis et al., 2019). Traditional computer programs utilize complex mathematical models and formulas to achieve automation and output of a series of schemes based on given programming models. This includes, for example, expert systems. At present, AI research has entered a distinct field of study known as machine learning (ML).

ML utilizes computational methods and data (experience) for training purposes. It does this to analyze the information that serves as the input and to process the information gained from accumulative experiences. The foundation of ML falls on “experience gathering” or “active learning.” In practice, this means that computers learn from input data and boost their properties by the mistakes they have made without specific programming or the establishment of a mathematical model (Erickson et al., 2017). In recent years, the rapid development of medical science means that it requires more precise and effective clinical treatment. Manual work increasingly has more disadvantages because of the need for high-density data processing. In parallel, mistakes are inevitable due to the inexperience of medical or dental professionals (Gao et al., 2019). The combination of ML and medical science, which is based on automatic operation workflow and the powerful operating capacity of computers, makes it possible to circumvent the constraints of manual work (Visvikis et al., 2019).

**Figure 1:**
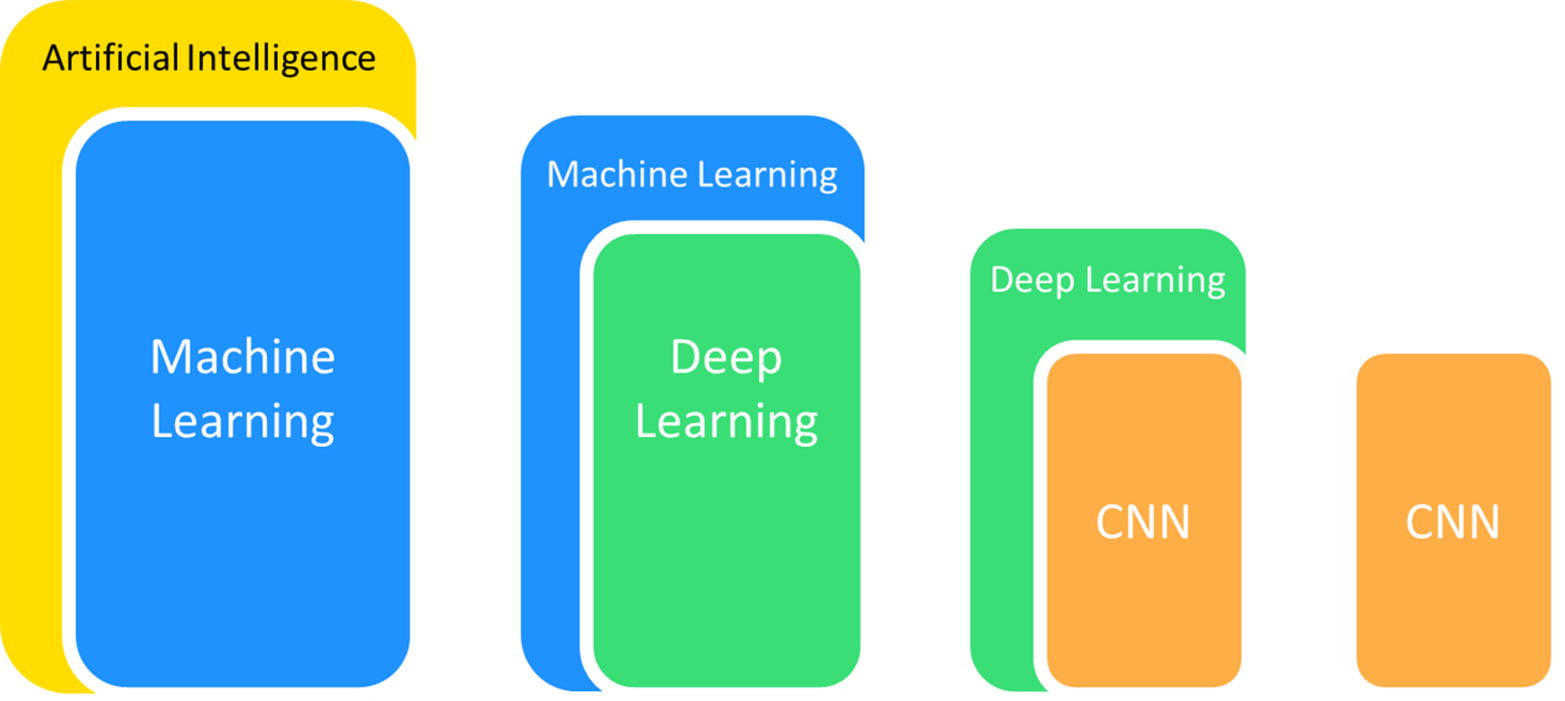
The popular branches of artificial intelligence used in medical imaging.

Computer-aided detection and diagnosis are two major domains of medical applications in ML (Gao et al., 2019). The application of algorithms for picture processing is ubiquitous in medical applications, particularly for the detailed analysis of medical images. These methods first carry out feature extraction of specific images and subsequently conduct target detection or categorize images into established classes to achieve image detection or classification. Convolutional neural networks (CNNs) have been applied the most frequently in medical imaging from the various ML models available because of their outstanding performance in disposing of image features (Lee et al., 2017). They offer outstanding performance in areas including radiographic recognition, analysis, segmentation and interpretation (Kulkarni et al., 2020) (Fig. 1). CNNs are thus used for pathological detection, diagnosis and prognosis.

Many studies have established a considerable number of ML models for use in medical and dental fields. Instances include the detection and evaluation of pulmonary nodules (Hung et al., 2020a) and diffuse lung diseases (Kido, Hirano & Mabu, 2020), diagnosis in dermatology, for example, for melanocytic lesions (Selim & Giovanni, 2019), as well as the segmentation of the prostate (Milletari, Navab & Ahmadi, 2016). Other examples include cancers like lung and breast cancer and show potential for diagnosis, detection and even prognostication using CNN (Gao et al., 2018; Hosny et al., 2018; Liu et al., 2018; Xie et al., 2019).

The applications of ML algorithms in dentistry and oral surgery are at early stages of development despite their potential as promising assistants for radiography (Schwendicke et al., 2019). In recent years, scientists have witnessed a drastic increase in research featuring dental, oral and craniofacial imaging with deep-learning methods, along with maxillofacial radiology and head and neck oncology. In this review, we first briefly demonstrate the working principle and rationale of ML in medical imaging. Second, we introduce the recent progress and applications of ML in dental radiography. Finally, we conclude the review with a summary of problems demanding prompt and timely investigation and resolution. We also describe our anticipations for the future research and development of AI in medical science.

### Why this review is needed and who it is intended for

ML is widely used in medical fields, including medical imaging and assisting clinicians in the diagnosis and treatment of disease. The application of ML in dental, oral and craniofacial imaging has been widely studied, and ML has been initially utilized in clinical treatment. These technologies have attracted the attention of dentists and are expected to become an important tool to assist in treatment. The combination of ML methods and medical imaging is a current trend and becomes increasingly necessary. In the years 2018–2020, around 30 articles have been published describing the application of deep learning, a subset of ML, to various fields of dentistry. The applications of ML in medical imaging have been reviewed by some researchers. However, no review is available to summarize in detail the applications of ML in the fields of dental, oral and craniofacial imaging, an area of much interest in dentistry as well as oral and maxillofacial surgery. Therefore, this review covers recent applications of ML methods in dental, oral and craniofacial imaging, points out problems that remain to be resolved and evaluates the prospects of the future development of this field of scientific study. The review is intended for dentists, oral and maxillofacial surgeons, other specialists and medical workers who are interested in AI.

## Survey methodology

We performed a systematic search of the literature in PubMed, Web of Science and IEEE ACM SPRINGER from 1980 to December 2020 to identify relevant articles for this review. The main free text and MESH terms used for our search can be divided into categories, and a combination of any words in different categories is applied to the search. At the same time, in order to achieve wider search results, we initially avoid adding specific types of data. Instead, we utilize the Boolean operator “NOT” to exclude unwanted results like studies using genomic data. The categories we used are as follows:

1. About ML: machine learning (ML); artificial intelligence (AI); neural network; convolutional neural network (CNN); support vector machine (SVM); regression; decision tree; random forest; deep learning; unsupervised learning; semi-supervised learning; fully convolutional network (FCN); U-net; ResNet; AlexNet; Lenet; DenseNet.
2. About imaging methods: radiography; cone beam computed tomography (CBCT); cephalometrics; X-ray; panoramic radiograph; lateral cephalogram; two-dimensional (2D); three-dimensional (3D); hyperspectral imaging; fluorescence imaging.
3. About oral cancer: oral cancer; head and neck cancer; head and neck squamous cell carcinoma (HNSCC); oral squamous cell carcinoma (OSCC); tongue cancer; oral tumor; detection; diagnosis; prognosis; survival rate.
4. About task and processing methods of ML: detection; prognosis; segmentation; object detection; classification.
5. About craniofacial imaging: orthodontics; landmark location; landmark annotation; superimposition; orthognathic surgery; dentofacial deformity.
6. About other dental diseases: dental caries; endodontic disease; periapical disease; periapical lesion; teeth extraction; periodontitis; root canal; dental pulp.

We only include the original research articles in the scope of references and single case reports were ruled out. We focus on the application of ML in dental, oral and craniofacial imaging, while studies with a wider range within the vague and broad concept of executive functions were excluded. This included the use of ML in other medical fields like respiratory diseases, other AI algorithms other than ML methods like expert systems, and other forms of data like clinical and genomic indicators. The preliminary research title and abstract were selected by four authors to determine whether they met the research criteria. We found about 540 relevant articles written in English through the preliminary search that may be useful for this review. We finally included about 170 studies that contributed to this review after reading the title and abstract of these articles.

## The working principle and rationale of ML

ML is a branch of AI. It can be seen from its name that it refers to the ability of machines to learn. ML is a general term for a class of algorithms that allow the machine to automatically dig out hidden laws from data, build models and then use the models to make decisions and complete other tasks. The core of these algorithms usually lies in data. The explosive growth in the amount of information that we witness today therefore gives these algorithms the vast soil upon which productive seeds can land and grow (Dey, 2016).

In the field of ML, four main learning methods exist: supervised learning, unsupervised learning, semi-supervised learning (weakly supervised learning) and reinforcement learning. Here we consider a task to be learned by a machine. Suppose there is a goal function “*G*”: *X*→*Y* that can accurately predict the output *Y* corresponding to each input *X*. This function is the ultimate goal learned by the algorithm. We can approximate the optimal solution to a certain extent, although it is impossible to find it. The method is to use a series of samples (*x*1, *y*1), (*x*2, *y*2),…,(*xi*, *yi*),…, (*xn*, *yn*) generated by the “Goal function” to estimate itself in order to put as many out-of-sample target pairs (*xj*, *yj*) in the function as possible. *xi* represents the feature we select to achieve the goal or the feature extracted by the algorithm, and *yi* represents the goal to be achieved by the task.

**Figure 2:**
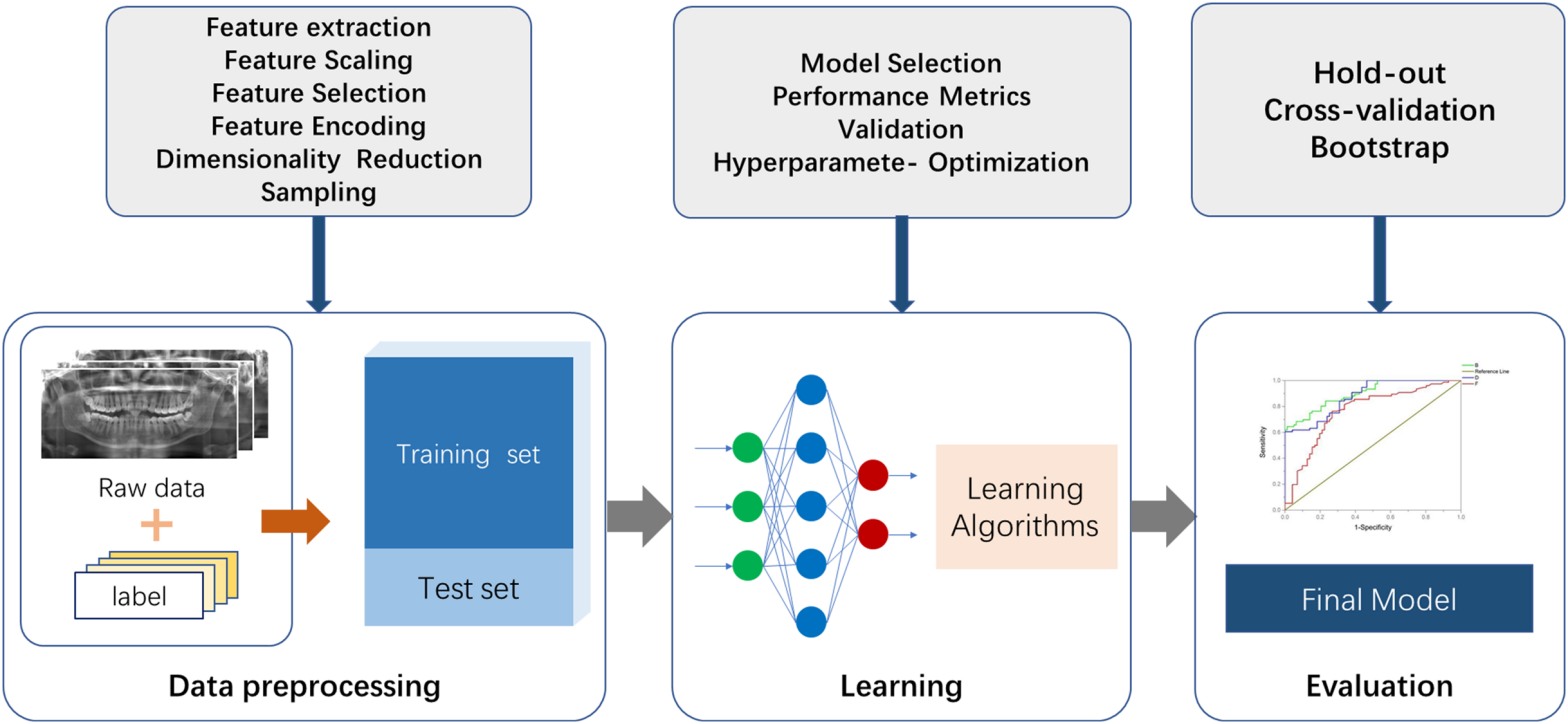
The fundamental machine learning procedure to achieve a final model.

There are two essential parts for the application of ML to actual scenarios. The first is data and features, and the second is models and algorithms. A commonly used ML procedure receives data that goes through preprocessing and manual labeling for training and testing. Selected ML algorithms are utilized for data learning, which occurs through model optimizing and model evaluation, and the mature model is finalized (Fig. 2). Specifically, the process of medical image processing typically consists of four steps: image acquisition, image pre-processing, image analysis and pattern recognition. The first step is image acquisition. The images processed in medical image processing are mostly acquired from medical imaging devices, including X-ray, CBCT and magnetic resonance imaging (MRI) (Taghanaki et al., 2021). The second step is to pre-process the images. Artifacts and noise on the images affect the image quality, which are due to the damage and contamination of the image caused by storage and transmission. Therefore, we need a series of image enhancement operations to recover or generate an image of the desired quality, including histogram equalization, image sharpening, thresholding transformations (Maini & Aggarwal, 2010) and various filtering operations. The third step consists of image analysis and feature engineering. This approach is used to extract the required features using a priori information and to send the image analysis results to the next stage of the ML model for training of the corresponding tasks. Shape features can be extracted by some boundary extraction operators including first-order differential Robert, Prewitt, Sobel operators, second-order differential Laplacian edge detection operators and optimal method-based operator (Canny, 1986). Spatial relationship features can be extracted by modeling pixel points, using methods such as Markov Random Field (Li, 1994). In image processing utilizing deep neural networks such as CNN, feature engineering can be performed using multi-layer convolution for adaptive extraction. This approach simplifies the amount of engineering, but specific feature information is difficult to extract and externalize. The fourth step is to feed the feature information extracted in step 3 into the selected ML model for modeling. Fallback steps such as hyperparameter adjustment and model adjustment are carried out based on the feedback from the results.

**Figure 3:**
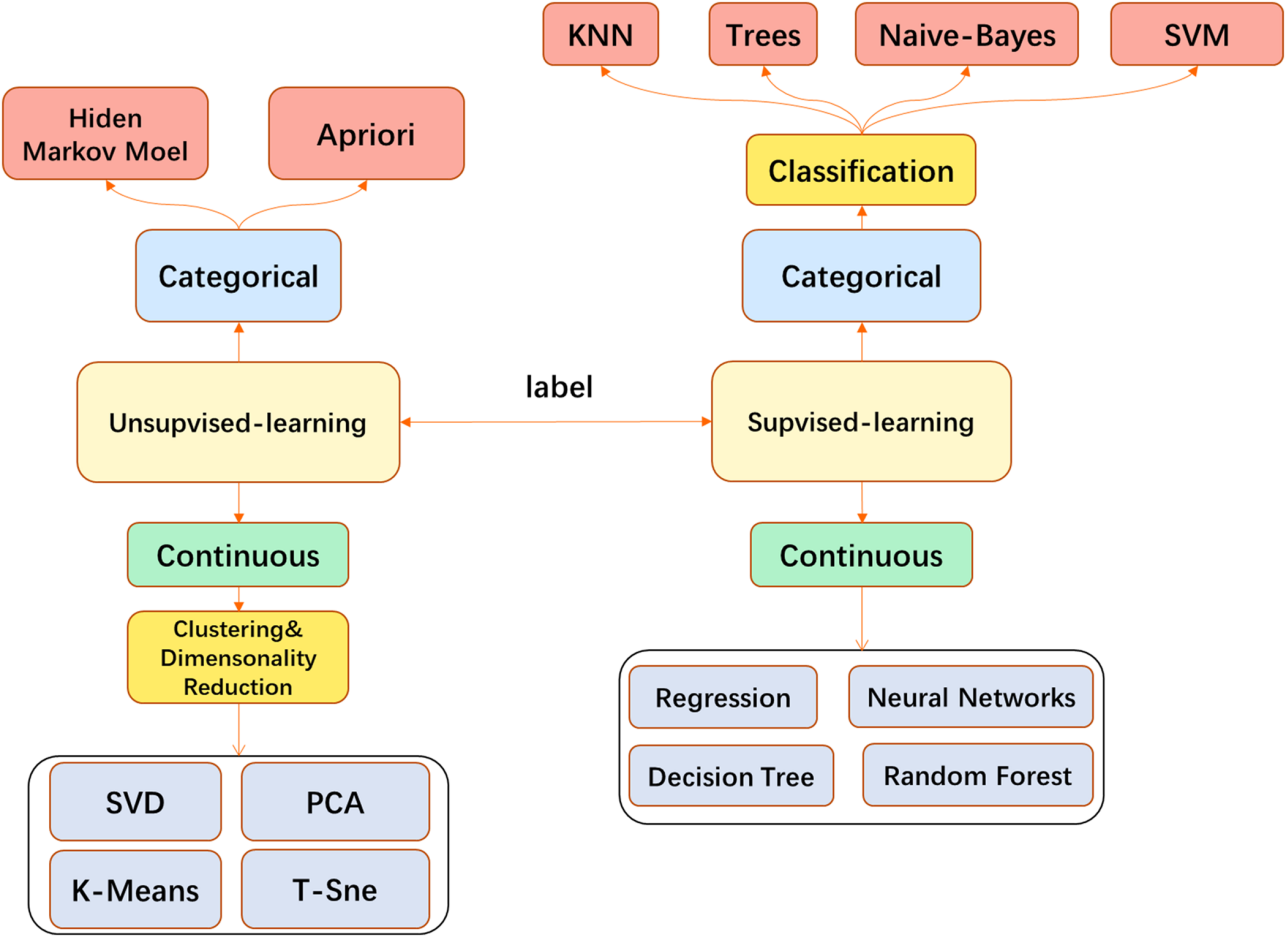
The main machine learning algorithms used in medical image processing.

In medical image processing, most traditional ML algorithms do not directly feed the original image into the model but go through some feature extraction processes. This may involve, for example, use of the well-known SIFT feature extraction analysis (Lowe, 2004) to obtain the features and send them to the model such as Support Vector Machine (SVM) (Smola & Schölkopf, 2004). Models such as k-Nearest Neighbor (KNN) (Cover & Hart, 1967) are used for tasks such as image classification or segmentation. When the CNN is finalized, the traditional feature engineering can be replaced by the convolutional layer and performed more efficiently. End-to-end task solutions can be realized, for example, and the convolutional layer with fully connected (FC) layers can be applied to image classification. CNN has become a popular model in the field of image processing and has been broadly used (Fig. 3).

CNN is an important type of neural network, and it is widely applied to image processing. The traditional method of FC layers, which existed before the advent of CNN, has been considered to have various disadvantages when used in image processing. Since it is constructed by how each neuron in the adjacent layer is linked, this architecture contains many weights (parameters in neuron networks), which dramatically increases the computational overhead. Compared to FC architecture, however, CNN is designed with some special characteristics.

The basis of CNN is a series of convolution operations that can be understood as the filter sliding on the image. A filter is a three-dimensional (channel, height, width) weight tensor that can extract the features from different pixel units. For a two-dimensional digital image, the affine transformation of the region can be realized after the filter is multiplied by the matrix of different regions of the image (Dumoulin & Visin, 2016). A filter is a collection of multiple different convolution kernels. A multiplying filter with the image area will yield responses of different magnitudes because of different kernels. Features are represented by the kernel in the area if the response is strong. The size of the filter is related to the receptive field, which represents the perception area of neurons (Hubel & Wiesel, 1962). It possesses a Gaussian distribution and also only takes up a small part of the full theoretical receptive field (Luo et al., 2016).

One kind of kernel usually corresponds to one specific pattern. Numerous different features can be extracted if filters with numerous different kernels are applied to an image. If there are kernels that correspond to identical patterns in a given image, the output of the convolution operation will respond strongly to those pixel units. These features include edges, directions and others. In addition, this operation largely reduces the number of weights because a filter conducts convolution operations for each pixel unit of the CNN architecture image. Furthermore, it means different image units can share the parameters, a process that is called parameter sharing (Ravanbakhsh, Schneider & Poczos, 2017). The use of kernels to process images can extract spatial information successfully, which means the interpretability of the parameters will be improved.

Feature maps are obtained after the convolution operation. However, the feature map size is still large even if the image has been processed by filters. A pooling layer has been proposed to further downsize the feature maps (LeCun et al., 1998). The pooling process is similar to the convolution operation, but its purpose is different: the filters used for pooling are usually designed to generate the maximum value or average value. The two methods involved in the pooling layer are called max-pooling and mean-pooling. They are usually used to extract the texture information and to collect the background information of feature points in the region (Boureau et al., 2010). The size of the feature maps can be reduced by pooling subsampling. It is therefore helpful to avoid overfitting and keep features robust against changes like rotation. Pooling subsampling can also reduce the calculation workload. However, these two pooling methods will cause excessive information loss and destroy the spatial information in processed images. Therefore, in order to compensate for the flaws of both pooling methods, researchers have made many improvements to them and presented methods like fractional max-pooling and others (Graham, 2014; Zeiler & Fergus, 2013; He et al., 2015). One trend is pronounced despite these improved pooling methods: many advanced networks are using fewer pooling layers and replacing them with convolution layers (Springenberg et al., 2014).

Problems remain to be solved after performing convolution and pooling operations to images. We have noted that these two operations are virtually continuous linear operations, and the output of the linear transformation superposition is another linear transformation. Therefore, by applying these two operations, we can only produce linear solutions and cannot handle indivisible linear problems. Nonlinear transformation is needed to compensate for the confinement of these two operations.

The solution to the aforementioned problem is activation functions, which are essentially nonlinear functions (Karlik & Olgac, 2011). The network can approximate any function according to the universal approximation theorem (Cybenko, 1989) when given sufficient linear output layers and nonlinear hidden layers. Only when a specific threshold value is achieved by the weighted sum of the signal intensity from previous dendrites will the subsequent neurons be activated in our nervous system for neurons in the biological sense. It is also necessary to discard some weak features for the neural network view, which is analogous to the biological neuron network, because it is unnecessary to store these features. The following are some well-known activation functions (Ramachandran, Zoph & Le, 2017): the softmax function, which is the earliest sigmoid function and the currently popular Relu function (Nair & Hinton, 2010). These two parts are the basic components of CNN. This is why a deep learning net is considered as a multi-stage distillation of information, where the information passes through continuous filters and is continuously purified.

In addition to these two fundamental parts, FC layers are often chosen to be a part of the network especially when networks are wanted to classify data (Krizhevsky, Sutskever & Hinton, 2012; Simonyan & Zisserman, 2014). Some unique variants have been well designed in order to adapt to other work. Fully Convolutional Network (Long, Shelhamer & Darrell, 2015) and U-net (Ronneberger, Fischer & Brox, 2015) are proven to be effective in semantic segmentation tasks. Yolov3 (Redmon & Farhadi, 2018) performs well in real-time object detection. In addition to these architecture changes, some optimization methods are also proposed in this process like Dropout (Hinton et al., 2012) and Adam (Kingma & Ba, 2014).

## Applications of ml in the dental, oral and craniofacial imaging field

Dental, oral and craniofacial imaging consists of several techniques from two-dimensions to three-dimensions. The most common imaging methods are CBCT and panoramic radiographs. Recent years have witnessed the burgeoning increase of the application of ML in this field. Our systematic search revealed that the usage of ML in the field of craniofacial imaging has become the biggest area of application, among which automatic cephalometrics has become relatively mature. In oral imaging, oral cancers, which are life threatening, have caught the attention of many researchers. Therefore, considerable studies focus on ML-based detection, diagnosis, prognosis and treatment design for these tumors, especially for OSCC, the oral cancer with the highest morbidity.

ML in craniofacial imaging has stepped into a multidirectional mature stage of research with many studies reported. Meanwhile, oral cancers are life-threatening diseases that cannot be easily diagnosed. Auxiliary diagnosis seems to be particularly meaningful. In addition, therapeutic schedules vary from person to person with different conditions of prognosis and process. The prediction of results based on ML may therefore lead to valuable references that improve the quality of life of patients. Other fields in oral medicine like endodontic and periodontal disease are likewise studied using ML approaches but mostly at the diagnostic level. In this section, we categorize the applications into three classes. First, we focus on the craniofacial imaging field, which includes orthodontics and orthognathic surgery. Second, we introduce the applications in oral tumors, covering their diagnosis, prognosis and the design of therapeutic regimen. Other applications will be grouped together.

### Application of ML in craniofacial imaging

#### Landmark location in cephalometrics

Automated cephalometric analysis is helpful in reducing the workload of orthodontists while achieving higher accuracy and efficiency (Dot et al., 2020). In 1984, computer-aided automated skeletal landmarking was created (Cohen, Ip & Linney, 1984). Today, various approaches have been used for cephalogram measurement. In the field of landmark location, the methods that have been most widely adopted into use can be roughly categorized into four branches: knowledge-based approaches (Gupta et al., 2015), model-based approaches (Romaniuk et al., 2004; Shahidi et al., 2014; Vucinic, Trpovski & Scepan, 2010), learning-based approaches (Kunz et al., 2020) and hybrid approaches (the combination of the first three approaches mentioned here) (Montúfar, Romero & Scougall-Vilchis, 2018). The first two approaches are considered deductive methods or analogical learning and are used to analyze radiographic structure via a defined set of patterns and models. Therefore, variability plays an important role in the final output data (Gupta et al., 2015), and both approaches are sensitive to image quality (Leonardi et al., 2008). By contrast, the recent widely applied approach of ML refers to learning by induction. Once training data are given, the computer produces the source concept itself based on a large dataset, which means it acts like a perception procedure.

Yue et al. presented a modified active shape model (ASM) to assist landmark location of lateral radiographs. This model is based on principal component analysis and grey pattern matching. Such an algorithm is built to capture variations of region shape and grey profile (Yue et al., 2006) by training with two hundred cephalograms of which 262 labeled feature points were set. The input pre-labeled images are marked by presetting 12 landmarks with good reliability. However, this method requires a large number of feature points to identify specific landmarks. The solution is to divide the whole lateral radiographs into smaller regions. The accuracy is highly relevant to the resolution ratio and initial position of the tested imagery graphs, which requires laborious work and has limitations in image quality.

Kunz et al. (2020) implemented cephalometric X-ray analysis by the application of a CNN mainly for landmark location. The customized CNN functioned as well as expert analysis, the golden standard for this type of study, after training with a total of 1,792 manually positioned lateral cephalometric radiographs. Numeric grey-scale values of each pixel, as input data, are recognized, and afterward the output layer acquires coordinate pairs of cephalometric landmarks after going through hidden layers with subsampling functions. Algorithms like You-Only-Look-Once version 3 (YOLOv3) network and Single Shot Multibox Detector (SSD) have been compared and analyzed in recent studies. YOLOv3 clearly outperformed SSD in time consumption and accuracy. In addition, no difference in detection error between YOLOv3 and manual landmark identification was found (Hwang et al., 2020; Park et al., 2019).

Two-dimensional radiographs lead to the deficiency of overall craniofacial morphology as well as information in the horizontal plane (Lenza et al., 2010). Other than traditional lateral cephalograms, CBCT imaging, which obtains details from the coronal, sagittal and horizontal positions, excels at lower radiation doses and where more structural information is present, and consequently is popular for dental imaging (Kiljunen et al., 2015). Many AI-aided types of research for cephalometric analysis work at the three-dimensional level (Gupta et al., 2016; Lee et al., 2019; Montúfar, Romero & Scougall-Vilchis, 2018; O’Neil et al., 2019).

Three-dimensional automated analysis is the ramification of plane cephalometrics. The main annotation methods can be classified into three categories: knowledge-based, atlas-based and learning-based methods (Dot et al., 2020). Gupta et al. (2015) created a knowledge-based algorithm in MATLAB that consists of preset mathematical entities. This approach works by finding the seed point, creating the volume of interest and extracting the contour of the valid skeletal structure. The corresponding landmarks on CBCT images are accessed by matching extracted contours with relevant mathematical entities. Furthermore, Montúfar, Romero & Scougall-Vilchis (2018) developed a hybrid method based on earlier work (Gupta et al., 2015) and a two-dimensional holistic ASM. The result suggests a potential role of the initial two-dimensional search algorithm in the improvement of accuracy and time saving for three-dimensional landmark annotation. Deep learning methods like CNN structure have also been conducted (Kang et al., 2020; Lee et al., 2019; Yun et al., 2020). Some structures like gonion, porion and others seem to be points with imperfect accuracy. In addition to algorithm insufficiency and manual errors, inexact anatomical positions and complex definitions are possible causes of this loss of accuracy (Ma et al., 2020; Montúfar, Romero & Scougall-Vilchis, 2018). However, only a few studies have been reported in the three-dimensional field of imaging, which suggests that it is still at the initial stages. Some research has produced tenable results, but further improvements are required to permit concrete conclusions.

One point worth noting is that the spatial landmark annotation can directly result from two-dimensional image learning. Lee et al. (2019) introduced a novel approach using shadowed two-dimensional image-based ML. VGG-Net is able to form stereoscopic craniofacial morphological structures after training using two-dimensional marked image data with different lighting angles and various views. A significant benefit of this approach is the reduction of input size. However, large errors persist in some landmarks. This approach offers new ideas, but many subsequent trials are needed.

#### Other branches in orthodontics

In addition to cephalometrics, the personalized design of orthodontic treatment is vital and significant.

Long-lasting therapeutic processes, optimal initiation times and optimal durations of orthodontic treatment are the main considerations for malocclusion types. Therapeutic interventions can help patients overcome the severity of different conditions and counter problems due to deficiencies in individual growth and development (Martonffy, 2015; Pinto et al., 2018; Pinto et al., 2017).

Orthodontists can better design the initial time of intervention by determination of the cervical vertebrae stages (CVS) from cephalometric radiographs (Chen et al., 2010; Uysal et al., 2006). Kök, Acilar & Izgi (2019) implemented a series of comparisons on CVS classifications using seven different AI algorithms, naming artificial neural networks (ANN) and evaluating other criteria. These algorithms analyze second to fourth cervical vertebrae and classify radiographs into six stages. The different stages are subsequently used to evaluate the decisions made for treatment time. ANN achieves the highest stability in a comparison of actual CVS with predicted CVS for the output of AI algorithms. ANN and SVM yielded the highest determination value in distinct stages of the area under the receiver operating characteristic curve (AUC) evaluation. More specifically, SVM achieves the highest accuracy in identifying CVS3 and CVS5, while ANN has the best performance in determining the other stages. SVM functions as a maximum margin classifier, maximizing the differences between disparate classes (Ben-Hur & Weston, 2010). Other evaluation methods also suggest that ANN displays both high relative accuracy and stability. ANN is therefore preferable in CVS determination. Recently, a study also compared the effectiveness of ANN with manual observation, and ANN was determined to be slightly inferior to human observers (Amasya et al., 2020). Other research described considerably high accuracy with ANN to achieve CVS evaluation (86.9% with 13 linear marks for each radiograph) (Kök, Izgi & Acilar, 2020). The differences may be due to measurement methods.

**Figure 4:**
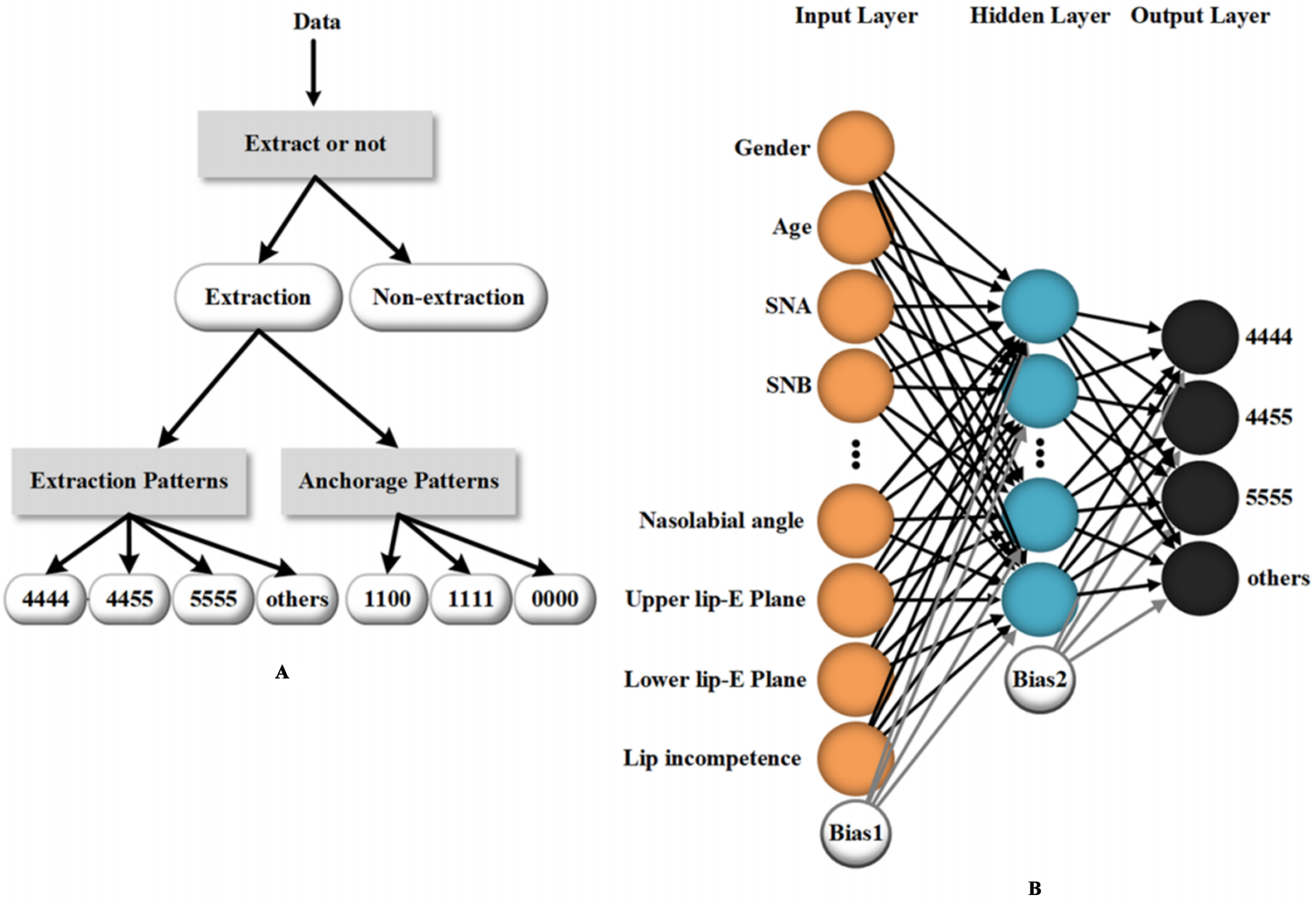
An example of a machine learning method (artificial neural network) utilized in orthodontic treatment design. (A) The data processing workflow of the artificial neural network which provides detailed guidance for the extraction and anchorage patterns. (B) The main inputting data and the structure of the three-layer neural network for tooth extraction prediction. Reprinted from (Li et al., 2019).

AI-assisted methods have been used in diverse ways in orthodontics. Some studies discuss the possibility of using ML to determine the necessity to extract teeth and the need for orthognathic-orthodontic surgery (Choi et al., 2019; Jung & Kim, 2016; Takada, Yagi & Horiguchi, 2009; Xie, Wang & Wang, 2010). Jung et al. (Jung & Kim, 2016) created a two-layer neural network to perform the extraction or non-extraction decision. The procedure sets four classifiers and consists of three stages: determining whether to extract teeth, the need for differential extraction between maxillae and mandible, and, eventually, the need for more retraction. The success rates of each stage tested were 93%, 89% and 84% (for more retraction with identical retraction) and 96% (for more retraction with differential extraction), which suggest a relatively high diagnostic precision. In addition to the need to extract teeth, the system of Jung provides a detailed plan of orthodontic treatment. In another study, ANN outputs detailed extraction patterns as well as anchorage patterns based on clinical and radiological data of orthodontic patients, which provides good treatment advice for orthodontists (Li et al., 2019) (Fig. 4).

In terms of poor image quality, which leads to unavoidable systematic errors, including noise and artifacts (particularly metal artifacts), the constraints can be reduced robustly and efficiently by deep learning methods (Huang et al., 2018; Jiang et al., 2018; Minnema et al., 2019; Zhang & Yu, 2018). Computer-assisted denoising and metal artifact reduction (MAR) succeed in improving the structural visualization and diagnostic accuracy of orthodontists, oncologists and doctors in other fields.

Orthodontists have accounted for a relatively large portion of users at present for the application of ML in oral medicine. Two-dimensional landmark location based on ML using traditional lateral cephalograms gradually shows promise. Multiple methods, especially various types of CNNs, produce good results.

The common use of CBCT means that cephalometrics is advancing to the three-dimensional stage. Three-dimensional cephalometrics has become a frontline direction for research. The need to retain more information means that landmark identification requires more suitable operations and specialized knowledge (Cevidanes, Styner & Proffit, 2006), which may be one of the principal obstacles to the automatic use of three-dimensional landmark annotation using AI methods. In ML, the lack of large training datasets might confine the development in three-dimensional fields because of ML features learning directly from data (Hwang et al., 2019). Other obstacles in the development of AI-supported applications in medical science include situations likely to take excessive amounts of time (computer learning time and manual cropping time) as many studies utilize manually preprocessed images for training data. Some other applications like CVS and orthodontic-orthognathic operation design also demonstrate the superiority of neural networks. The practicability of picture processing is therefore paving the way for the development of automatic orthodontic treatment. More mature clinical uses like image superimposition, detailed surgical procedure design and process simulation of orthodontic treatments can be achieved fully automatically with the help of ML methods.

#### Orthognathic surgery and other dentofacial deformities

In the field of orthognathic surgery, the use of ML can enhance the accuracy of diagnosis from maxillofacial images (Sun et al., 2018; Zamora et al., 2012), assist in customizing the computer-aided design and manufacture (CAD/CAM) of orthodontic and surgical appliances and equipment (Cevidanes, Styner & Proffit, 2006) and can be improved by comparing the results at finer intervals through image superposition (Bouletreau et al., 2019).

Hyuk-Il Choi et al. (Choi et al., 2019) developed a ML model by studying 316 samples. Twelve lateral cephalogram measurements and six additional indexes were used as input for the model to calculate the success rate of surgical decision-making. Patcas et al. (2019a) have shown that ML can be used to evaluate the facial attractiveness and appearance age of orthognathic patients. Patcas et al. (2019b) evaluated the facial attractiveness of the forehead and side images of ten patients with a left cleft lip and ten controls using a special convolution neural network, and concluded that the ML method can be a powerful tool to describe facial attractiveness. Facial symmetry is an important indicator of facial attractiveness. Lin et al. used a novel Xception model to score facial symmetry before and after orthognathic surgery. Special two-dimensional contour maps converted from CBCTs were considered as the input data. These maps contain much three-dimensional information (Lin et al., 2021). Jeong et al. studied the front and side faces of more than 800 subjects with dentofacial dysmorphosis/malocclusion using CNNs and found that CNNs are able to relatively accurately estimate the soft tissue contours related to orthognathic surgery with these photographs alone (Jeong et al., 2020). However, as far as the current results are concerned, important adjustments need to be made to the ML model. CBCT images combined with ML models can also be used to measure the bone mineral density of the implant area (Çolak, 2019; Dahiya et al., 2018), evaluate the bone mass of the surgical area (Suttapreyasri, Suapear & Leepong, 2018) and assist in the construction of a static guide plate system (Lin et al., 2020).

AI passage from two-dimensional to three-dimensional imagery along with the added benefits of increased diagnostic precision make the treatment effect visual and the communication between doctors and patients unimpeded. However, the value and ability of ML in simulating the consequences of orthognathic surgery have not been fully proved. Bone displacement will make it difficult to predict soft tissue changes. The response displacement of soft tissue to the underlying bone can vary greatly according to the mass, and there are many influencing factors. An algorithm is still unlikely to accurately predict the final aesthetic effect after surgery.

### Application in oral cancers

Oral cavity cancer is a high-risk category of life-threatening tumors and accounts for the major proportion of head and neck cancer (Rivera, 2015). In addition to several functional symptoms like teeth loss, head-neck pain and potentially fatal consequences, this craniofacial disease also likely results in disfigurement of patients without early diagnosis or favorable prognosis. Classical oral cancer detection and diagnosis are based on radiological analysis, clinical monitoring indicators and histopathological assessments (Mahmood et al., 2020). Prevention and early-stage diagnosis are of great significance to the survival rate and treatment management of cancerous patients. However, the definite tumor diagnosis is usually late (Chakraborty, Natarajan & Mukherjee, 2019; Rivera, 2015). In recent years, conventional and modern ML methods, especially neural networks and SVM, have illustrated the capability of processing oral cavity tumor-related image data. This includes oral cancer detection and tissue cell classification in the stage of cancer diagnosis (Al-Ma’aitah & AlZubi, 2018; Aubreville et al., 2017; Das, Hussain & Mahanta, 2020; Jeyaraj & Samuel Nadar, 2019; Shamim et al., 2019), tumor margin assessment and tumor subtype classification in the process of clinical cancer treatment (Fei et al., 2017; Marsden et al., 2020; van Rooij et al., 2019) and assessment of complications after treatment (Ariji et al., 2019; Dong et al., 2018; Men et al., 2019). Major tumors like OSCC are able to be detected and evaluated with high accuracy using a timesaving algorithm (Aubreville et al., 2017; Das, Hussain & Mahanta, 2020).

#### Detection of oral cancers

Semantic image segmentation and feature extraction are two fundamental processes of image classification through ML methods. They form the basis of oral cancer detection by this type of approach (Haider et al., 2020; Mahmood et al., 2020). Hyperspectral Imaging (HSI) is a currently applicable technique for tumor detection. HSI, which contains three-dimensional data, provides a potential noninvasive approach to assess pathological tissue by illustrating spectral features of different tissue (Akbari et al., 2011; Lu et al., 2014). Pandia et al. (Jeyaraj & Samuel Nadar, 2019) established a deep CNN, which is used in the classification and evaluation of hyperspectral cancerous images. The researchers extract image features at the first stage using a weight-based technique, and a two-layer partitioned, regression-based deep CNN classifier is employed subsequently for feature classification. The discrimination accuracy using an expert classification scheme between malignant and benign tumors reaches 91.4%, while the accuracy between malignant tumors and precancerous lesions reaches 91.56%.

Chan et al. (2019) designed a two-branch deep CNN method for oral cancer detection and localization. Original auto-fluorescence images were chosen and disposed of to output texture maps. Afterward, the specific texture maps are utilized by ML to conduct automatic localization of cancer. The Gobar filter, which is used to implement image feature extraction, achieves detection sensitivity and specificity of 93.14% and 94.75%, respectively.

Oral leukoplakia is the most common type of precancerous lesions of oral cancer. A study by Jurczyszyn utilizes intraoral photographs to conduct oral leukoplakia prediction, which can be considered for early prevention of oral cancers (Jurczyszyn, Gedrange & Kozakiewicz, 2020). However, oral cancer consists of a large variety of distinct malignancies. Hence, primarily distinguishing tumor-related tissue from imaging data of patients is fundamental and essential but lacks precision for specific oral cancers.

Some other studies have focused on the diagnosis of single oral cancers (Aubreville et al., 2017; Das, Hussain & Mahanta, 2020; Rahman et al., 2020; Shamim et al., 2019). Squamous cell carcinoma is responsible for approximately 90% of total oral cancers and has become the sixth most common cancer worldwide (D’Souza & Addepalli, 2018; Kar et al., 2020).

Biopsy is the current gold standard for OSCC (Swinson et al., 2006), but the histopathological method is time-consuming and costly. Therefore, Navarun et al. (Das, Hussain & Mahanta, 2020) utilize four types of deep CNN models through a transfer learning approach, and one proposed CNN to achieve the automated histological grading of whole slide images on lesion locations.

Transfer learning can reduce the amount of training data as it fine-tunes from the previously trained large dataset. Biopsy is invasive and painful for patients, and some researchers are looking for ways for noninvasive imaging. Confocal Laser Endomicroscopy (CLE) imaging has proved capable and reliable in the detection of HNSCC (Nathan et al., 2014). It has been used by Marc and coworkers (Aubreville et al., 2017) for OSCC microstructure assessment.

The researchers collected both normal tissue images from the alveolar ridge, inner labium, hard palate and cancer-related tissue images as samples. A binary classification (normal or cancerous) with an accuracy of 88.3% is obtained using CNN. The real-time identification instrument is of use for automated detection of cancerous lesions.

Carcinogenic factors need to be taken into consideration. Infection by human papillomavirus (HPV) is one of the high-risk factors for OSCC (Marur et al., 2010). ML-based cancer detection also succeeds in evaluating molecular markers like HPV. Some studies use contrast computed tomography (CT) for capturing features of HPV-related head and neck squamous cell carcinomas (Huang et al., 2019; Zhu et al., 2019). MRI data are also utilized for OSCC assessment. Specific MRI texture features, which are chosen by dimension reduction, are capable of automatic OSCC histological grading without biopsy (Ren et al., 2020). The grading accuracy of OSCC achieves an average of nearly 85% using three types of classifiers. It is useful to assess histological grading via MRI since it is a noninvasive approach for clinical examination.

Trials on automated screening of other oral cavity cancers have been implemented clinically (Shamim et al., 2019) or on experimental animals (Lu et al., 2018). Six deep CNNs have been applied to distinguish tongue lesions before tongue cancer fully takes hold (Shamim et al., 2019). The VGG19 model demonstrates the best capability in classifying benign and pre-cancerous lesions using original resized photographic images as input, while the ResNet50 model shows its potential in the discrimination of five lesion subtypes. Researchers have further combined computational outcomes with physician decisions and increased the binary classification accuracy to 100%. At the same time, some benign tumors in the oral cavity are also detected automatically by some ML methods, which includes ameloblastoma, keratocystic odontogenic tumor, pleomorphic adenoma, Warthin tumor and others (Poedjiastoeti & Suebnukarn, 2018; Al Ajmi et al., 2018). These tumors also make up a large proportion of oral tumors.

The majority of these studies are related to cancerous image classification, and most studies have achieved desirable detection accuracies compared to the gold standard. ML has yet to reach the needed precision for tumor diagnosis. The automated detection methods, which are based on a series of diverse imaging approaches, improve clinical cancerous workflow and provide assistance for the decisions of oncologists. However, a lack of training datasets and data quality restrict the scale of research. More image data with the standard format are required for future research.

#### Clinical treatment of oral cancers

Morphological analysis, including tumor margin assessment and tumor site evaluation, is of much concern during the treatment of oral cancers. The tumor size and site are connected with prognosis (Chakrabarti et al., 2010; Namin et al., 2020), for example, for patient survival rate and surgical decisions for tumor resection (Upile et al., 2007). Much research has focused on the automated structure segmentation of oral cancer-related images (Brouwer de Koning et al., 2018; Fei et al., 2017; Grillone et al., 2017; Marsden et al., 2020; van Rooij et al., 2019). In a study by Fei et al. (Fei et al., 2017), hyperspectral images of surgical cancerous tissue samples have been acquired to train and test the ML model. The principle of this method is also related to tissue classification, through which the margins of oral tumors are profiled clearly with an average accuracy of 90%. Additionally, the research also compares the impact of image types on the precision of margin assessment, which turns out to outperform HSI over fluorescence images.

The use of real-time oral cavity screening probes with ML methods has also been reported for surgical procedures (Marsden et al., 2020). Fluorescence lifetime imaging (FLIm) is a noninvasive technique capable of assessment of molecular composition (Cheng et al., 2013). Mark and coworkers (Marsden et al., 2020) utilized and compared three ML models to conduct both in vivo and ex vivo tumor margin assessment. They used fiber probes to acquire oral tissue specimens, and further processed the tissue regions with different classifiers: SVM, Random Forests and CNN. “Cancer,” “Health” and “Dysplasia” labels were annotated on the scanning images after the visualization process using Python. The outcomes demonstrate the potential of FLIm to predict pre-cancerous tissue and suggest that the Random Forest technique is superior to the other two popular image-processing methods.

#### Prognosis of oral cancers

Post chemoradiotherapy complications of cancers are severe and individualized. In addition to common side effects like myelosuppression, osteoradionecrosis and hair loss, specific complications after chemoradiotherapy include xerostomia, hearing loss, inflammation of skin and mucosa and cancer recurrence (Haider et al., 2020). Kuo and coworkers (Men et al., 2019) collected CT scan data from patients undergoing radiation therapy and developed a prognostic system using three-dimensional residual CNN (3DrCNN) to predict the occurrence of post-therapy xerostomia. The implementation of the 3DrCNN method is followed by structural segmentation, which outlines margins of parotid and submandibular glands on CT scans. Radiation dose distributions, profiles of salivary glands and CT scans are prepared as optional data, and at least two of them are selected as input. The model without data rejection reaches the best performance with accuracy, sensitivity and specificity of all around 0.76. The worst performance occurs when the radiation dose label is lacking. Further studies can focus on the accuracy of methods for structure identification and to increase the data types for input (e.g., treatment cycle) to augment the precision of xerostomia prediction.

Five-year survival rate and survival time of cancer patients are significant indicators for cancer prognosis, as well as references for therapeutic outcomes. In a recent study, a total of 59 patients with oral tongue cancer have been examined (Pan et al., 2020). All were treated with radiotherapy, and their CT images utilized and studied by computer for individual survival prediction.

The researchers used a t-Distributed Stochastic Neighbor Embedding (t-SNE) method to screen out effective features to allow for numerous irrelevant features. Probabilistic Genetic Algorithm-Back Propagation (PGA-BP) ML methods were used, and the prediction accuracy was already close to actual survival conditions: 30.5 ± 21.3 months for actual survival time and 31.6 ± 15.8 months for predicted survival times. Furthermore, to improve the degree of accuracy, other indicators including tumor grading and staging should be taken into account. The year of diagnosis, the age at diagnosis and cancer size and site are of significance in the lifetime of patients (Hung et al., 2020b).

Oral malignancies have a close relation with cervical lymphatic metastasis, which implicates poor cancer prognosis (Okada et al., 2003; Spiro, 1985) and is especially indicative of the sharp decrease in the 5-year survival rate (Exarchos, Goletsis & Fotiadis, 2012; Taghavi & Yazdi, 2015; Walk & Weed, 2011). Therefore, the detection of metastasis for cervical lymph nodes has become a focus of attention after clinical treatment. In this context, automated detection with the help of ML methods has been conducted with distinct image types recently (Ariji et al., 2019; Dong et al., 2018; Keek et al., 2020). The nodal status of oral cavity SCC and oropharyngeal SCC is assessed using contrast-enhanced CT scans. The bagging of Naïve Bayes achieves the best accuracy of 92.9% with receiver operating characteristic (ROC) of 0.857 (Romeo et al., 2020). Additionally, more than 10,000 contrast-enhanced CT images of cervical lymph nodes have been trained by CNN, and the analytical result suggests a close precision for evaluation between manual and automated assessment.

In another study (Dong et al., 2018), the assessment sensitivity based on a non-radiating thermal system was higher than that based on contrast-enhanced CT scans, but the two pieces of research utilize distinct ML models.

The wiser choice of image types for assessment needs further comparison. MRI has also been considered as a potential predictive modality for HNSCC prognosis (Yuan et al., 2019). In future, computer-assistant methods can be explored and put into application. One type of research utilizes both MRI image features and clinical information such as smoking history, age and other factors to automatically predict the existence of HPV in patients suffering from oropharyngeal squamous cell carcinoma (Bos et al., 2021). Relations between clinical characteristics and HPV status are also analyzed as the existence of HPV is closely related to cancer prognosis. Others have also estimated the existence of HPV and *p53* mutation in HNSCC patients via MRI (Dang et al., 2015; Ravanelli et al., 2018).

Automated oral cancer detections and assessments are available for diverse image data, most of which are CT scans and HSI (hue, saturation, intensity).

CNN models achieve high-quality cancer-related image processing and are mainly used as a means of image classification, especially functioning as binary classifiers. Other ML methods like SVM and Random Forests also display high sensitivity, accuracy and specificity during image processing with specific types of image data. However, their superiority needs further exploration and more evidence due to the lack of sufficient research literature. Meanwhile, given that a large amount of recent research utilizes limited imaging data for AI training, the significance of data sharing and dataset construction is highlighted (Haider et al., 2020).

The combination of AI and molecular imaging has aroused attention with the rapid progress in ML-based imaging. Rather than conventional automated imaging, which works by image classification, applications at the level of molecular imaging place emphasis on biomarker exploration (Choi, 2018). Biomarkers of oral cancers may assist in unambiguous tumor detection and individualized treatment. At the genetic level, the complex genomic data can be extracted by ML methods effectively, which demonstrates a distinct way for the detection of oral cancer and its evaluation (Chang et al., 2013; Li et al., 2017). SVM shows promising capabilities in genomic studies. Future directions of research will include AI-related, imaging–genomic combined studies to enhance analytical effectiveness and the accuracy of oral cancers.

### Other fields in dental, oral and craniofacial imaging

ML is widely used in the field of stomatology. It has important clinical value, including but not restricted to the detection of dental caries, periapical disease, periodontology, facial recognition, the evaluation of facial attractiveness evaluation and other uses.

Hong Guofeng and coworkers (Hung et al., 2020a) established a non-clinical caries detection model and realized caries detection by obtaining and analyzing two-dimensional images of extracted teeth. You et al. (2020) studied plaque detection of deciduous teeth based on deep learning. Schwendicke et al. (2020a) applied deep CNN to detect caries in near-infrared transparent (NILT) images, and they also emphasized that applying AI for caries detection is less costly and more effective (Schwendicke et al., 2020b). Zhang X et al. developed ConvNet, which is based on CNN, to identify dental caries from oral images captured with consumer cameras. The result showed that the image-wise sensitivity is good (Zhang et al., 2020). At present, more studies have been conducted on the possibility and accuracy of AI-assisted detection of dental caries, but there are few studies on AI-assisted prediction of the occurrence of dental caries. The automatic detection of superficial dental caries is also a problem that needs a solution.

ML is increasingly being used by both dentists and researchers as a novel method for diagnosing dental diseases, especially endodontic diseases. The condition of teeth is a significant factor of influence for stomatognathic system health. The classification and segmentation of tooth and root canal by ML methods has achieved promising results both at the two-dimensional and three-dimensional levels (Dumont et al., 2020; Zhang et al., 2018). Dumont et al. (2020) combined an intraoral scanner-acquired crown form with a CBCT-obtained root form to realize the clinical labeling of spatial crown and root morphology. Image segmentation work was conducted using both U-Net and ResNet. The follow-up clinical diagnosis or therapeutic schedule can be more complete and more precise once the tooth morphology has been ascertained.

Different studies (Fukuda et al., 2019; Hiraiwa et al., 2019; Orhan et al., 2020) have utilized a CNN system to detect the root morphology, longitudinal root fissure and periapical lesions of molars. Lee, Kim & Jeong (2020) used CNN-based dental panoramic X-rays and CBCT images to detect and diagnose three types of odontogenic cystic lesions as follows: odontogenic keratocyst, odontogenic cyst and periapical cyst. They also developed a computer-aided detection system using a deep CNN algorithm (Lee et al., 2018), which was found to be useful in PCT diagnosis and provided predictable evaluation of periodontal injury. Early osteoarthritis can also be automatically detected by providing radiologic, clinical and molecular level information for the temporomandibular joint (Bianchi et al., 2020). Most of the studies are in the initial stages with insufficient clinical applications.

We conclude that the research of image detection based on CNN has been intense in the past few years. One of the development trends of AI in oral imaging research in the future will be using CNN to combine image detection with clinical treatment and further advance smarter decision making for medical treatments.

ML models have also proved useful in other clinical application domains. Examples include the following: the diagnosis of maxillary sinusitis (Ohashi et al., 2016), the classification of third molar developmental phase and tooth type (Tuzoff et al., 2019), the classification of periapical slices, the identification of dental plaque and gingival inflammation at the root canal opening, the automatic allocation of dental age estimation and the diagnosis of multiple dental diseases (Benyo, 2012; Hwang et al., 2019). The major applications of ML in dental, oral and craniofacial imaging are summarized in Table 1.

**Table 1:** Applications of ML methods in dental, oral and craniofacial imaging.

Fields	Subfields	Types of ML	Researches
Orthodontics	Landmark identification	Active shape model (ASM)	The algorithm functions by capturing variations of region shape and grey profile, based on segmentation of lateral cephalograms. High imagery quality and tedious works are needed (Yue et al., 2006)
		Customized open-source CNN deep learning algorithm (Keras & Google Tensorflow)	Study uses high quality training data for supervised learning. With a huge set of 1792 lateral cephalograms, the algorithm demonstrates comparable precision with experienced examiners (Kunz et al., 2020)
		You-Only-Look-Once version 3 (YOLOv3)	The study uses 1028 cephalograms as training data, which consists of both hard and soft tissue landmarks. The mean detection errors are not clinically significant between AI and manual examination. Reproducibility seems better than manual identification (Hwang et al., 2020; Park et al., 2019)
		Hybrid: 2D active shape model (ASM) & 3D knowledge-based models	The study uses a holistic ASM search to get initial 2D cephalogram projections. Further it utilizes 3D approaches for landmark identification. With the preprocessing of 2D algorithms, the accuracy and speed of landmark annotation are heightened (Montúfar, Romero & Scougall-Vilchis, 2018)
		Entire image-based CNN, patch-based CNN & variational autoencoder	With only a small amount of CT data, the hierarchical method (consists of 4 steps) reaches higher accuracy than former researches on 3D landmark annotation with deep learning methods. The mean point-to-point error is 3.63 mm (Yun et al., 2020)
		VGG-Net	The study has trained VGG-Net with a large amount of diverse shadowed 2D images. Each image has different lighting and shooting angles. The VGG-Net is able to form stereoscopic craniofacial morphological structure (Lee et al., 2019)
	determination of cervical vertebrae stages (CVS)	k-nearest neighbors (k-NN), Naive Bayes (NB), decision tree (Tree), artificial neural networks (ANN), support vector machine (SVM), random forest (RF), and logistic regression (Log.Regr.)	The study suggests that the seven AI algorithms have different precision of determination. ANN reaches the highest stability, the lowest accuracy occurs in Log.Regr. and kNN. By overall consideration, ANN is recommended to CVS determination (Kök, Acilar & Izgi, 2019)
	Teeth-extraction decision	A two-layer neural network	The process consists of three steps: initial determination of teeth extraction, the choice of differential extraction, and determination of specific teeth to be extracted. The neural network gives a detailed plan of teeth extraction in orthodontic treatment (Jung & Kim, 2016)
Oral cancer	Detection of oral cancers	Texture-map based branch-collaborative network	Deep CNN is used for cancer detection as well as localization, the detection sensitivity and specificity achieve 93.14% and 94.75% respectively (Chan et al., 2019)
		Alexnet, VGG-16, VGG-19, Resnet-50, & a proposed CNN	The study utilizes five CNNs for automated OSCC grading. The proposed CNN performs best with accuracy of 97.5% (Das, Hussain & Mahanta, 2020)
		Regression-based deep CNN with 2 partitioned layers, Google Net Inception V3 CNN architecture	The deep learning method is implemented on hyperspectral images, with the amount of training data growing from 100 to 500, the tissue classification accuracy (benign or cancerous) increases by 4.5% (Jeyaraj & Samuel Nadar, 2019)
	Cancer margin assessment	SVM, Random Forests, 6-layer 1-D CNN	Fiber probes are utilized to collect FLIm data with ML methods. Random Forest demonstrates best performance in tissue region division (healthy, benign and cancerous tissue), displaying potential in tumor surgical visualization (Marsden et al., 2020)
	Prognosis of oral cancer	3-D residual CNN (rCNN)	The study uses three types of labels as inputting data: CT images, radiotherapy dose distribution and contours of oral cancers. And the rCNN model is able to extract features on CT images to predict post-therapeutic xerostomias with best accuracy of 76% (Men et al., 2019)
		Deep learning method, AlexNet architecture	The system is implemented on contrast-enhanced CT to assess cervical lymph node metastasis in patients carrying oral cancers. The diagnostic results demonstrate little differences between manual and automated evaluation (Ariji et al., 2019)
		back propagation (BP), Genetic Algorithm-Back Propagation (GA-BP), Probabilistic Genetic Algorithm-Back Propagation (PGA-BP) neural networks	Three ML approaches are utilized for cancerous patients’ survivial time prediction. PGA-BP has the best performance with an error of of average survival time for less than 2 years (Pan et al., 2020)
Dental endodontics	Detection of dental caries	CNN, the basic DeepLab network, DeepLabV3+ model	The dental plaque detection model was trained using natural photos based on a CNN framework and transfer learning. Photos of deciduous teeth before and after the usage of a dental plaque exposure agent were used. Results show that the AI model is more accurate (You et al., 2020)
	Root morphology	CNN, the standard DIGITS algorithm	This study analyzed CBCT and panoramic radiographs of mandibular first molars with a total of 760. The root image block is segmented and applied by deep learning system. High accuracy in the differential diagnosis of distal root forms of the mandibular first molar (single or multiple) was observed (Hiraiwa et al., 2019)
	Periapical lesions	deep CNN	CBCT images of 153 periapical lesions were evaluated by deep CNN, and it was able to detect 142 periapical lesions, which is capable to figure out the location and volume of lesions and detect periapical pathosis based on CBCT images (Orhan et al., 2020)
		The deep learning approach based on a U-Net architecture	This study achieved periapical lesions detections by segmenting CBCT images. The accuracy of DLS lesion detection reaches 0.93 (Setzer et al., 2020)
	Periodontology	CNN, the GoogLeNet Inception-v3 architecture	The study utilized panoramic and CBCT images to detect three types of odontogenic cystic lesions (OCLs) based on CNN and transfer learning. Results suggest that CBCT-based training performs better than panoramic image-based training (Lee, Kim & Jeong, 2020)
		deep CNN architecture and a self-trained network	The study utilized deep CNN algorithm for periodontally compromised teeth (PCT) diagnosis and prediction. The accuracy of PCT diagnosis on premolars reaches high level than that on molars (Lee et al., 2018)
Orthognathic surgery	facial attractiveness	CNN, VGG-16 architecture	The study viewed the photos of 146 orthognathic patients before and after treatment, assessed their facial attractiveness and apparent age using CNN, and found that the appearance of most patients improved after treatment (Patcas et al., 2019a)
		CNN, VGG-16 architecture	Full-face and lateral pictures of left cleft lip patients and controls were assessed and facial attractiveness was evaluated. Results showed that CNN is capable to assess facial attractiveness with similar score of manual evaluation (Patcas et al., 2019b)
	Others	CNN	CBCT images combined with AI can also be used to measure the bone mineral density of the implant area, evaluate the bone mass of the surgical area, and assist in the construction of static guide plate system (Dahiya et al., 2018; Lin et al., 2020; Suttapreyasri, Suapear & Leepong, 2018)

## Concluding remarks

In recent years, ML has gradually penetrated all fields of dentistry, most of which are related to teeth and, in some cases, to gums and dental tissue, dental arch, osteoporosis and others. In our review, we mostly focus on applications in the fields of craniofacial imaging and oral cancer. ML, especially CNNs, not only helps doctors to screen diseases quickly but also assists in diagnosis and treatment. However, some aspects need to be supplemented to promote the sustainable development of oral and maxillofacial radiology deep-learning research. First, the data needed for these types of research are internal, which results in difficulties in making objective comparisons and poses great needs for professional labeling. Second, the imbalance in the quality of medical records quality, some good and some bad, makes it difficult to conduct studies with big data. In addition, the insufficient number of data is another obstacle. Recent research with ML usually has only less than 1,000 X-rays in each group. For three-dimensional data like CBCT, sometimes less than 100 units are used for training. Third, because of the large amount of computation and long training time, higher requirements exist for computer hardware (Schwendicke et al., 2019). Fourth and finally, deep learning cannot be completely intellectualized. It must rely on considerable existing data samples in order to analyze and predict new data, but for disease analysis, we often cannot control the variability of this data (Ruellas et al., 2016).

The development of ML has faced major challenges. However, significant progress has been made for ML applications in current trials such as two-dimensional landmark annotation and detection of oral diseases. Some of these applications have obtained the same high accuracy as the current golden standard or achieved even better results. The image-processing capability of ML has particular significance in oral medicine. However, many applications are at an incipient stage and are far from clinical use. In view of data deficiencies, efforts can be made to transform learning models: for example, turning the complex, integral model to several modularized structures, modifying previous ML models to handle multifarious, accessible data or adopting the method that combines manual operation with computer assistance. The production of more high-quality medical imaging data sets in the future will also greatly promote this direction of research. Novel semi-supervised and weak-supervised algorithms will also help alleviate this problem. In addition to model design, the operating cost also needs to be taken into consideration. Overall, ML has a bright future as a way to increase clinical efficiency and diagnostic accuracy.

There are three types of main tasks in medical imaging: image classification, image positioning and detection and image segmentation. Good progress has been made in all three fields, especially in the areas of medical image registration and segmentation. However, application has been weak, and more innovative work is needed. Some theoretical and application aspects of machine learning applied to medical imaging require improvement. In addition, new theoretical breakthroughs and creative applications are needed to promote the further development of this direction. In particular, many theoretical areas need significant advances. For example, the application of three-dimensional convolution to medical imaging can compensate for the three-dimensional features of organ tissues that cannot be extracted by traditional two-dimensional convolution (Zhu et al., 2018). Three-dimensional reconstruction technology can help visualize the internal structure of the human body, which enables surgical navigation and early-stage auxiliary diagnosis (Dong et al., 2018). A multi-modal information extraction method can synthesize information obtained by different devices to generate more accurate information. In addition, a time series model can be used to generate metastasis trajectory predictions for oral tumors and other diseases.

ML is likely to be more widely used in medical imaging as current trends in medical development gather pace, and we witness the inevitable convergence of the medical and computer sciences,. In the future, many of the existing barriers for medical image-assisted diagnosis technology will be overcome by the application of ML as its accuracy improves. Further effort should ensure a smarter and more effective imaging analysis in the fields of dental, oral and craniofacial research.

## Additional Information and Declarations

### Data Availability

The following information was supplied regarding data availability: This is a literature review.
